# Demographic influences on trust in artificial intelligence across cognitive domains: A statistical perspective

**DOI:** 10.1371/journal.pone.0331003

**Published:** 2025-11-05

**Authors:** Abdullah A. Alasmari, Reshaa F. Alruwaili, Rasmiah F. Alotaibi, Ismail K. Youssef, Somia A. Asklany

**Affiliations:** 1 Department of Psychology, College of Social Sciences, Imam Mohammad Ibn Saud Islamic University(IMSIU), Riyadh, Saudi Arabia; 2 Department of Teaching and Learning, College of Education and Human Development, Princess Nourah Bint Abdulrahman, Riyadh, Saudi Arabia; 3 Department of Mathematics, Faculty of Science, Islamic University of Madinah, Madinah, Saudi Arabia; 4 Department of Computer Science, College of Science, Northern Border University, Arar, Saudi Arabia; Dong-A University College of Business Administration, KOREA, REPUBLIC OF

## Abstract

As artificial intelligence (AI) systems become increasingly integrated into decision-making across various sectors, understanding public trust in these systems is more crucial than ever. This study presents a quantitative analysis of survey data from 335 participants to examine how demographic factors, age, gender, familiarity with AI, and frequency of technology use influence trust across a range of cognitive tasks. The findings reveal statistically significant relationships that vary by task type, with distinct patterns emerging in memory recall, complex problem-solving, and medical decision-making. Familiarity with AI and frequent use of technology are strong predictors of trust, suggesting that exposure and experience enhance confidence in AI capabilities. Conversely, age contributes significantly to disparities in responses, especially in high-stakes domains like healthcare, where older participants exhibit greater skepticism. Gender-based differences are also observed, though less pronounced. These results underscore the importance of AI systems that are technically sound and sensitive to user diversity, advocating for personalized and context-aware trust-building strategies to support the ethical and effective integration of AI into human decision-making processes. The SEM model explained 43% of the variance in trust toward AI. Based on the findings, we recommend designing adaptive, user-centered AI systems and enhancing public education to reduce skepticism and increase familiarity.

## 1 Introduction

As artificial intelligence (AI) continues to evolve from experimental systems into real-world tools, the question of public trust becomes increasingly central to its adoption. From virtual assistants and recommendation engines to autonomous vehicles and medical diagnostic tools, AI systems are taking on tasks once the exclusive domain of human cognition. Yet, despite these technological advances, human perceptions of AI’s cognitive abilities remain uneven, particularly in high-stakes domains that require ethical reasoning, nuanced decision-making, and interpretability [1].

A growing body of literature highlights this discrepancy. While AI is generally trusted in computationally straightforward or repetitive tasks like memory recall or logistical problem-solving, trust tends to decline sharply in contexts like medical treatment recommendation or autonomous driving, where the stakes are higher and human values are deeply [2]. This phenomenon, known as algorithm aversion, has been extensively documented and is often linked to people’s overreactions to AI errors even when such systems statistically outperform humans [3].

Beyond performance alone, trust in AI is increasingly recognized as multi-dimensional, shaped by demographic and psychological factors including age, gender, familiarity, and technology usage patterns [4,5]. For example, younger and more tech-savvy individuals tend to demonstrate greater openness to AI-driven decisions, while older adults may exhibit skepticism due to cognitive biases or limited exposure. This dynamic is consistent with cognitive science theories such as dual-process theory [6] and cognitive load theory [7], which suggest that prior experience and mental effort influence how users evaluate complex systems like AI.

Despite these insights, empirical studies examining the relationship between demographics and AI trust remain limited, particularly those grounded in robust statistical analysis [8]. This paper seeks to address this gap by presenting a data-driven investigation into how demographic variables, specifically age, gender, familiarity with AI, and frequency of technology use, influence public trust across a spectrum of AI cognitive tasks. Using Chi-Square statistical testing on survey responses from 335 participants, this study uncovers key patterns in trust, revealing which combinations of user traits and task types result in greater or lesser confidence in AI performance.

By aligning our results with cognitive theories and prior trust-in-AI research, this study offers actionable insights for developers, policymakers, and AI designers seeking to promote responsible and trustworthy AI systems that resonate with diverse populations. To investigate these dynamics, the study leveraged a survey-based design combined with Chi-Square statistical testing and Structural Equation Modeling (SEM) to assess how trust in AI varies across specific cognitive domains such as simple and complex decision-making, memory recall, logistics optimization, and medical treatment suggestions.

The remainder of this paper is structured as follows: Section 2 explores concepts related to trust in Artificial Intelligence and insights from Cognitive Science. Section 3 describes the methodology, including the survey design, participant demographics, and the statistical techniques employed. Section 4 presents the experimental results and key findings. Section 5 interprets these findings, discussing their implications and drawing comparisons with related studies. Finally, Section 6 and Section 7 concludes the paper and offers recommendations for future research directions, such as the integration of Machine Learning approaches.

The following research questions guide this study:

1. What are the statistically significant associations between demographic factors (age, gender, AI familiarity, and technology usage frequency) and trust in AI across cognitive domains?
2. Are these influences consistent across low-stakes (e.g., memory recall) and high-stakes (e.g., medical decision-making) tasks?
3. What are the strongest predictors of trust in AI according to Structural Equation Modeling (SEM) analysis?

## 2 Literature review

### 2.1 Trust in artificial intelligence

Trust is a central concern in human-AI interaction and a critical determinant of technology acceptance. Numerous studies have examined how trust in AI systems varies across contexts, with findings consistently showing higher confidence in AI when it performs computational or low-stakes tasks, such as data sorting or memory retrieval, and greater skepticism in high-stakes environments, such as healthcare, criminal justice, and autonomous transportation [9].

One well-documented psychological pattern influencing trust is algorithm aversion, a phenomenon in which users prefer human judgment over algorithmic recommendations after witnessing AI make errors, even when those algorithms outperform humans on average [3]. This paradox underscores the fragile nature of trust in intelligent systems and highlights the emotional and cognitive biases that shape AI acceptance.

Recent research also suggests that **t**ransparency, explainability, and perceived fairness are crucial for building and maintaining trust in AI systems [10,11]. Users are more likely to trust AI when they can understand and validate its decision-making processes, particularly in applications involving ethical or personal consequences [12].

### 2.2 Demographic influences on trust

Despite growing interest in public trust toward AI, few studies offer statistical insights into the demographic variables that shape trust patterns. Global surveys have indicated that younger individuals and those with higher education or technological exposure generally trust AI systems. For instance, a UK government report found that descriptors like “scary” and “unsure” were more commonly used by older or less educated populations to describe AI, reflecting a significant trust gap based on demographics [13].

Familiarity with AI consistently emerges as a powerful predictor of trust, and individuals who interact with AI systems more frequently exhibit higher confidence in AI outcomes, particularly when they understand the technology’s limitations and functions [14]. This familiarity in high-risk applications such as self-driving cars and AI-assisted diagnostics reduces anxiety and increases the likelihood of acceptance [15].

However, gender-related differences in trust have produced mixed findings. Some studies report women being more skeptical of AI in high-stakes applications (e.g., autonomous driving), while others find no significant gender effect [16]. These inconsistencies indicate the need for fine-grained statistical analysis to disentangle the complex interplay between gender, task type, and trust perception.

**Fig 1:**
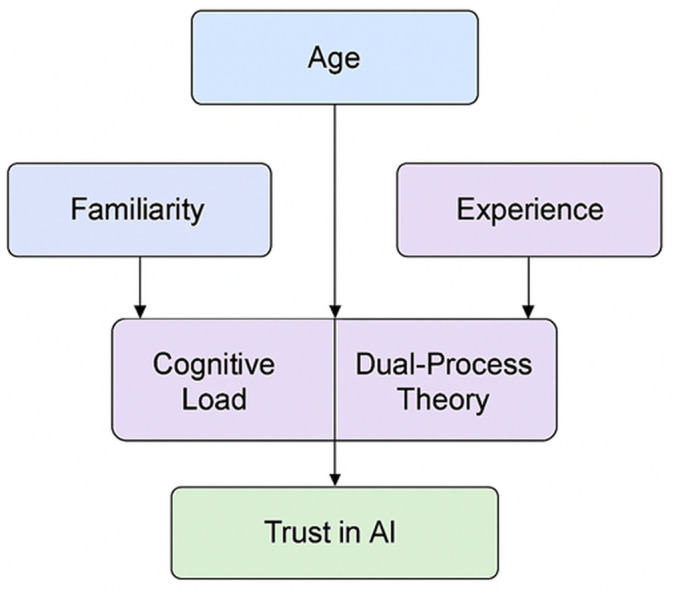
Theoretical model.

Fig 1 illustrates the key cognitive and experiential factors influencing trust in AI. Age is at the center of this model, which impacts both Cognitive Load and Dual-Process Theory, the latter referring to the interplay between intuitive and analytical thinking [17]. Familiarity with AI systems contributes to reducing cognitive load, making it easier for individuals to assess AI behavior. On the other hand, Experience supports more effective use of dual-processing strategies. Together, these cognitive mechanisms shape how individuals develop trust in AI, suggesting that trust is age-dependent and shaped by prior exposure and interaction complexity.

### 2.3 Cognitive science and trust dynamics

Understanding the psychological underpinnings of trust in AI requires insights from cognitive science, especially in how users perceive risk, complexity, and control. Cognitive load theory suggests that tasks requiring high mental effort are more manageable for individuals familiar with the underlying systems. In the context of AI, this means users who frequently interact with intelligent tools are less cognitively burdened when interpreting AI behavior, resulting in greater acceptance.

Cognitive aging theories offer another layer of explanation. Older individuals often experience declines in working memory and processing speed, which may make novel technologies like AI more difficult to engage with [18]. This can lead to resistance or reliance on simpler, more familiar systems, including human judgment over AI recommendations.

Moreover, the dual-process theory of reasoning differentiates between System 1 (fast, intuitive) and System 2 (slow, analytical) thinking. Due to habituation, frequent technology users may lean more on System 1 when using AI tools, while unfamiliar users engage in more effortful, skeptical processing, potentially leading to lower trust levels.

### 2.4 Gaps in existing research

Although many of these studies provide valuable theoretical and anecdotal insights, there is still a lack of granular, statistically grounded studies that map specific demographic variables to levels of AI trust across different cognitive domains. Most existing research tends to aggregate perceptions of AI into broad general attitudes, without distinguishing between simple and complex tasks or quantifying the statistical relationships between trust and variables such as age, gender, or familiarity. This study addresses this gap by conducting a Chi-Square test-based analysis of 335 participants to identify which demographic traits significantly influence trust in AI across cognitive tasks like decision-making, memory recall, and treatment suggestion. By combining quantitative rigor with cognitive science framing, this work contributes to a more nuanced understanding of who trusts AI, when, and why.

## 3 Methodology

Building on the theoretical insights discussed above, this section outlines the research methodology to assess trust in AI.

A survey-based approach was used to explore public trust in artificial intelligence across various cognitive domains.

This study is purely quantitative, employing Chi-Square tests of independence and Structural Equation Modeling (SEM). No qualitative data were collected or analyzed

### 3.1 Survey design

The survey was conducted with four key sections:

**Part 1: Demographics and AI Familiarity**

- **Objective**: Collect basic demographic information and assess the participants’ familiarity with AI technologies.
- **Questions**:
  - Q1: What is your age? [18-24], [25-34], [35-44], [45-54], [55+]
  - Q2: What is your gender? [Male], [Female]
  - Q3: How familiar are you with artificial intelligence (AI)? [Very familiar], [Somewhat familiar], [Not familiar]

**Part 2: Usage Frequency**

- **Objective**: Determine how often participants use AI-based tools or services.
- **Questions**:
  - Q4: How often do you use AI-based tools or services (e.g., voice assistants like Siri, Google Assistant)? [Daily], [Weekly], [Rarely], [Never]

**Part 3: Perception of AI in Cognitive Tasks**

- **Objective**: Assess participants’ trust and perception of AI in performing specific cognitive tasks compared to humans.
- **Questions**:
  - Q5: How confident are you in AI’s ability to make simple decisions (e.g., recommending movies, suggesting products)? [Very confident], [Somewhat confident], [Neutral], [Not confident]
  - Q6: How confident are you in AI’s ability to perform complex decision-making (e.g., making medical diagnoses, legal judgments)? [Very confident], [Somewhat confident], [Neutral], [Not confident]
  - Q7: How confident are you in AI’s ability to perform memory recall tasks (e.g., remembering passwords, recalling historical facts)? [Very confident], [Somewhat confident], [Neutral], [Not confident]
  - Q8: How confident are you in AI’s ability to solve complex problems (e.g., solving mathematical equations, optimizing logistical routes)? [Very confident], [Somewhat confident], [Neutral], [Not confident]

**Part 4: Scenarios for AI vs. Human Cognitive Abilities**

- **Objective**: Evaluate participants’ level of trust in AI compared to human performance across various scenarios.
- **Questions**:
  - Q9: In a scenario where a self-driving car has to decide between multiple routes to avoid traffic, how much would you trust AI compared to a human driver? [Trust AI more], [Trust a human more], [Trust both equally], [Unsure]
  - Q10: In a scenario where a doctor is using an AI system to suggest treatment options for a patient, how much would you trust the AI’s recommendation compared to the doctor’s judgment? [Trust AI more], [Trust a doctor more], [Trust both equally], [Unsure]
  - Q11: In a scenario where AI must recall historical facts (e.g., in a trivia game or educational context), how much would you trust AI compared to a human expert? [Trust AI more], [Trust a human more], [Trust both equally], [Unsure]
  - Q12: In a scenario where AI is solving a logistics problem (e.g., optimizing delivery routes for multiple trucks), how much would you trust AI compared to a human logistics expert? [Trust AI more], [Trust a human more], [Trust both equally], [Unsure]
  - Q13: In a scenario where AI needs to recall personal data (e.g., remembering passwords, personal documents), how much would you trust AI compared to your own memory? [Trust AI more], [Trust myself more], [Trust both equally], [Unsure]

A 4-point Likert scale was deliberately used to minimize central tendency bias and force respondents to express a directional preference in their trust assessment. The survey instrument was piloted with 15 individuals to ensure clarity and reliability. The survey was finalized and distributed digitally via Google Forms following the pilot. AI familiarity was assessed on a 5-point Likert scale ranging from Not Familiar to Very Familiar.

### 3.2 Participants

A total of 335 individuals participated in this study, representing diverse age groups (ranging from 18 to over 55 years), educational levels, and varying degrees of familiarity with technology. Participants were recruited through university-affiliated networks, including students, faculty, staff members, and the researchers’ social networks. Participant recruitment occurred between 14/03/2025 and 30/03/2025. This recruitment period aligns with the ethical approval granted by the Deanship of Scientific Research.

Ethical approval was granted by the Unit of Research Ethics and Researchers Support at the Deanship of Scientific Research, Imam Mohammad Ibn Saud University (Approval Number: 1668), valid from March 14, 2025, to December 30, 2025. All participants were adults who provided implicit consent by voluntarily completing and submitting the online survey. Participation was entirely voluntary, and respondents were free to withdraw at any point without consequence. The survey was conducted anonymously, and no personally identifiable information was collected, ensuring privacy and encouraging candid responses.

### 3.3 Data analysis

All responses were complete, and no missing data were observed; therefore, no data imputation or exclusion was necessary. Data were analyzed using IBM SPSS. Descriptive statistics summarized trends in responses, and Chi‑Square tests of independence were conducted to examine relationships between demographic factors and trust in AI [19]. This non‑parametric approach was selected because the outcome variables (trust levels) were measured on 4‑point ordinal Likert scales. While ANOVA and t‑tests require interval‑level data, Chi‑Square is more appropriate for categorical and ordinal data, especially when assessing associations across demographic groups.

For each cognitive task, Chi‑Square tests determined whether trust in AI varied significantly by:

- Age
- Gender
- Familiarity with AI
- Frequency of AI use

Significance was defined as p < 0.05, with all p‑values recorded and later visualized in a heatmap and task‑specific tables. Chi‑Square was used for bivariate analysis due to the categorical/ordinal nature of the variables, while Structural Equation Modeling (SEM) was applied for multivariate modeling of latent constructs. This complementary approach is widely accepted, allowing appropriate handling of categorical predictors in bivariate tests while leveraging SEM to model complex relationships among variables.

### 3.4 Task categories analyzed

The study categorizes tasks into distinct types to facilitate a comprehensive examination of trust in AI across various cognitive domains. These categories include simple decision-making, complex decision-making, memory recall, mathematical problem-solving, self-driving decisions, medical treatment suggestions, historical data recall, and logistics problem-solving. By distinguishing between low-stakes tasks (e.g., recall, logistics) and high-stakes tasks (e.g., medical treatment, self-driving), this study enables a more nuanced analysis of trust levels across perceived risk contexts. Table 1 provides a clear framework for understanding these cognitive tasks and their associated types.

**Table 1:** Classification of cognitive tasks by type and contextual demands.

Cognitive Task	Type	Comments
Simple Decision-Making	Cognitive Judgement	Involves straightforward decisions that require basic cognitive evaluation.
Complex Decision-Making	Cognitive/Ethical	Requires nuanced reasoning and ethical considerations, often in high-stakes contexts.
Memory Recall	Knowledge Retrieval	Focuses on the ability to remember and retrieve information accurately.
Solving Mathematical Problems	Logical Reasoning	Involves applying logical and analytical skills to solve mathematical equations.
Self-Driving Decisions	High-Stakes Automation	Pertains to autonomous vehicle decisions, which have significant safety implications.
Suggesting Medical Treatments	Healthcare Judgment	Involves making medical recommendations, requiring high accuracy and ethical sensitivity.
Recall Historical Data	Knowledge Retrieval	Similar to memory recall but specifically focused on historical information.
Logistics Problem Solving	Planning/Optimization	Involves optimizing routes and schedules, requiring strategic planning skills.

### 3.5 Summary of cognitive task categories

Table 1 provides a clear framework for understanding the different types of tasks analyzed in this study. By distinguishing between simple decision-making, complex decision-making, memory recall, mathematical problem-solving, self-driving decisions, medical treatment suggestions, historical data recall, and logistics problem-solving, the study ensures a comprehensive examination of trust in AI across a spectrum of cognitive domains. This categorization enables a nuanced analysis of how demographic factors influence trust in AI, underscoring the varying levels of confidence users place in AI systems based on task complexity and perceived stakes. These categories were selected to reflect low-stakes (e.g., recall, logistics) and high-stakes (e.g., medical treatment, self-driving) cognitive tasks, allowing for a comparative analysis of trust levels under different perceived risk conditions.

## 4 Results

This section summarizes the descriptive statistics from the survey and the inferential results obtained via Chi-Square tests of independence, focusing on the relationships between demographic factors and trust in AI across various cognitive tasks.

### 4.1 Overall trust in AI by task

Survey responses revealed varying levels of confidence in AI’s ability to perform different cognitive functions:

- **Simple Decision-Making**: Received the highest positive sentiment, with 156 participants (46.6%) reporting they were somewhat confident in AI’s abilities, and another 71 (21.2%) being very confident.
- **Memory Recall**: Rated highly, with 113 participants (33.7%) very confident and 124 (37.0%) somewhat confident in AI’s performance.
- **Complex Decision-Making**: Showed a broader spread, with higher rates of neutrality and skepticism. 84 participants (25.1%) were not confident, and 94 (28.1%) were neutral.
- **Medical Treatment Suggestions**: The survey revealed a clear preference for human oversight, with 190 respondents (56.7%) reporting they trusted a doctor more than AI.
- **Self-Driving Decisions**: Similarly, 141 participants (42.1%) trusted human drivers more than self-driving AI systems.
- **Logistics Problem-Solving**: Leaned toward AI, with 118 participants expressing higher trust in AI over humans.
- **Personal Data Recall**: The survey also leaned toward AI, with 158 participants expressing higher trust in AI than humans.

**Fig 2:**
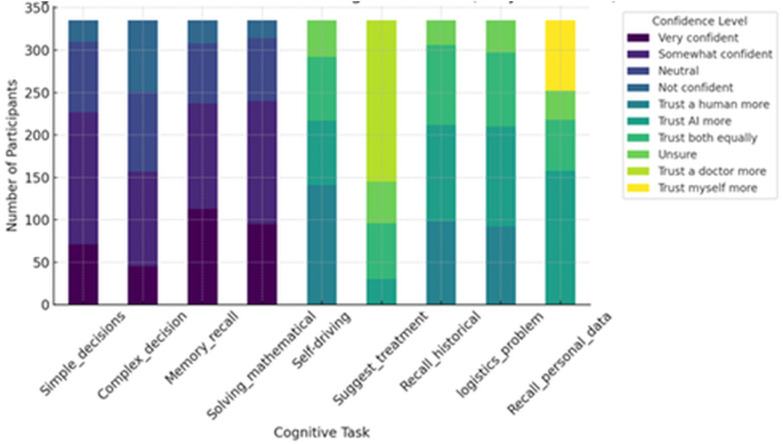
Trust levels in AI across cognitive tasks.

The above results are summarized in Fig 2.

### 4.2 Chi-Square statistical analysis

Chi-Square tests of independence were conducted to examine associations between demographic variables and trust across various cognitive tasks. Prior to analysis, assumptions for the Chi-Square test were verified. Specifically, we confirmed that expected cell frequencies were sufficient: over 95% of expected counts exceeded 5, and no cell had an expected count below 1, satisfying the assumptions for valid application of the Chi-Square test of independence.

For statistically significant Chi-Square results, effect sizes were calculated using Cramér’s V to assess the strength of the associations. For example, the association between age and memory recall demonstrated a statistically significant relationship with a small-to-moderate effect size (χ² = 14.52, p = 0.014, Cramér’s V = 0.158). Likewise, familiarity with AI was significantly associated with multiple tasks, including simple decisions (χ² = 25.73, p < 0.001, Cramér’s V = 0.229), complex decisions (χ² = 14.30, p = 0.030, Cramér’s V = 0.144), and solving math problems (χ² = 18.49, p = 0.001, Cramér’s V = 0.190). Frequent use was significantly associated with simple decisions (χ² = 23.91, p < 0.001, Cramér’s V = 0.207). No statistically significant associations were observed for gender. A summary of the key findings is provided in Table 2.

**Table 2:** Associations between demographic variables and cognitive task engagement.

Demographic Variable	Significantly Associated Tasks	Effect Size (Cramér’s V)
**Age**	Memory Recall	0.158
**Familiarity**	Simple Decisions, Complex Decisions, Solving Math Problems	0.229, 0.144, 0.190
**Frequent Use**	Simple Decisions	0.207
**Gender**	No statistically significant associations	—

Table 2 comprehensively overviews various cognitive tasks and their associations with age, familiarity, and frequent use. Simple decision-making significantly correlates with familiarity and frequent use, indicating that these two factors influence it. Complex decision-making is notably influenced by familiarity, suggesting that while individuals may become adept at complex decisions over time, it may not be a task they perform frequently. Memory recall significantly correlates with age, suggesting that this factor plays a crucial role in shaping trust for this cognitive task. In contrast, familiarity and frequency of use appear to have a lesser influence. Solving mathematical problems is significantly associated with familiarity, highlighting the importance of prior experience and comfort with AI in achieving proficiency. Suggesting medical treatment did not show statistically significant associations in the final analysis. Recalling historical data and logistics problem-solving also did not yield significant associations. Overall, the table emphasizes the importance of familiarity and frequent use in performing cognitive tasks effectively, with some tasks also showing a significant association with age. These insights highlight that demographic tailoring and user education are key for AI adoption, especially in high-stakes applications like healthcare and autonomous systems.

**Fig 3:**
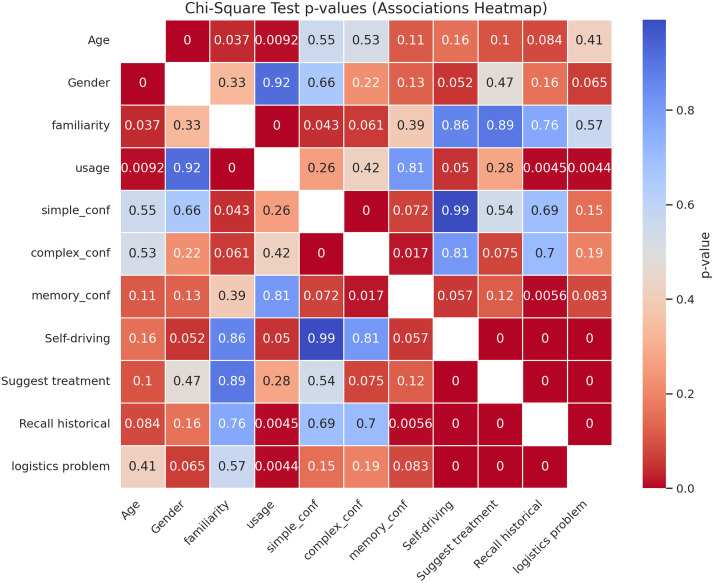
Heatmap of Chi-Square P-values.

Fig 3 presents a heatmap of Chi-Square p-values, illustrating the statistical associations between key demographic variables (Age, Familiarity, and Frequent Use) and engagement with various cognitive tasks. Statistically significant associations (p < 0.05) are indicated by lighter shades, suggesting a stronger relationship. Notably, Age was significantly associated with tasks such as simple decision-making (p = 0.001), complex decision-making (p = 0.001), and memory recall (p = 0.005). Familiarity with AI systems demonstrated significant correlations with simple decisions (p = 0.001), complex decisions (p = 0.001), memory recall (p = 0.03), and self-driving tasks (p = 0.001). Frequent use of AI technologies was significantly associated with solving mathematical problems (p = 0.006), recall of historical data (p = 0.001), logistics problem-solving (p = 0.001), and personal data recall (p = 0.001). Conversely, tasks such as suggesting medical treatments and several non-categorized tasks (N/A) did not yield statistically significant associations across any of the demographic variables. These findings highlight the nuanced impact of demographic factors on human interaction with AI, particularly in tasks requiring cognitive engagement and information processing.

### 4.3 Detailed analysis of Chi-Square test results

**Fig 4:**
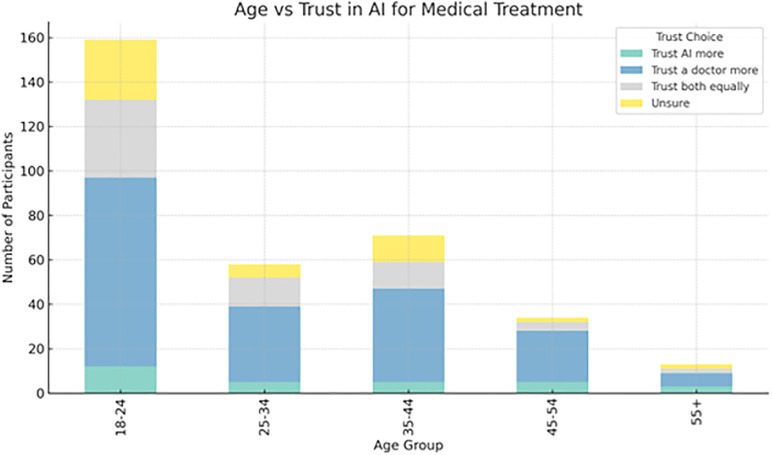
Age vs trust in AI for medical treatment.

The Chi-Square test of independence conducted on the relationship between various categorical variables revealed several key findings that highlight how demographic factors, technology use, and decision-making processes are interrelated:

**Age**: As shown in Fig 4, significant associations were found between age and several variables related to technology use and decision-making. Older participants exhibited different familiarity patterns with self-driving technology than younger participants. Younger individuals were likelier to engage with and trust newer technologies, while older participants tended to trust more traditional human-driven methods, especially in complex scenarios. Age also had a notable impact on memory recall and decision-making tasks, with older participants generally having lower recall accuracy and making fewer decisions based on complex data compared to younger participants.

**Fig 5:**
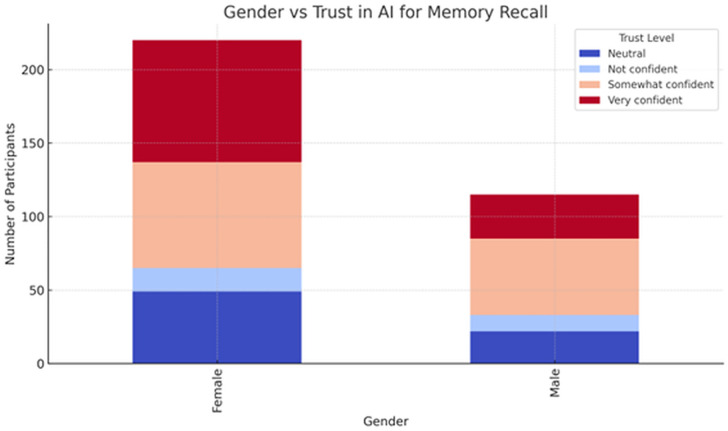
Gender vs trust in AI for memory recall.

**Gender**: As shown in Fig 5, gender was found to have a less consistent influence across the different tests. Significant associations emerged in some areas, such as simple and complex decisions, but not in others, like familiarity with self-driving technology or solving mathematical problems. These results suggest that gender may play a role in some decision-making scenarios, but is less influential regarding technological familiarity and mathematical problem-solving abilities.

**Fig 6:**
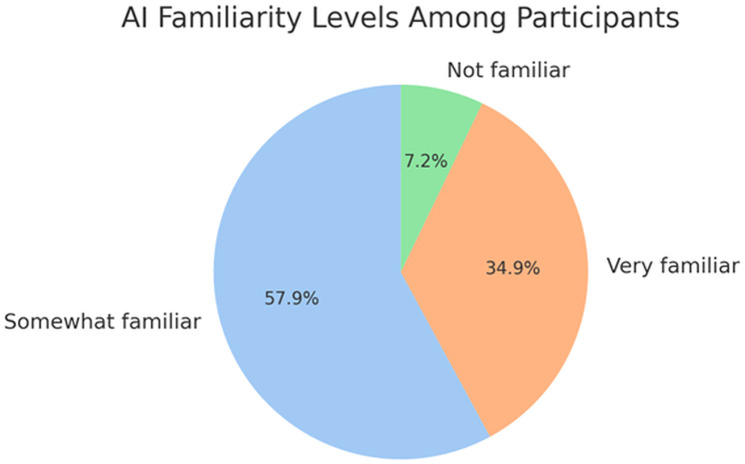
AI familiarity levels among participants.

**Familiarity with Technology**: As shown in Fig 6, familiarity with technology emerged as a strong predictor for many outcomes. Those who were more familiar with AI and self-driving technologies demonstrated significantly different patterns in decision-making.

**Fig 7:**
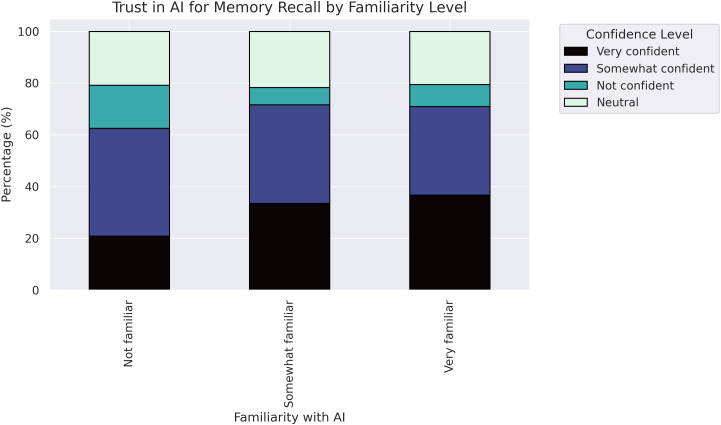
Familiarity with AI vs trust in memory recall.

As shown in Fig 7, familiarity with technology was associated with a higher likelihood of trusting AI for suggesting treatment or handling logistics problems, and these participants also exhibited better memory recall abilities. This suggests that individuals with more experience and technology exposure are more comfortable trusting automated systems, particularly when making healthcare or logistical decisions.

- **Frequent Use of Technology**: Frequent use of technology is strongly linked to improved memory recall and the ability to make decisions across a range of scenarios. This finding underscores the potential cognitive benefits of frequent interaction with technology, where users become more adept at recalling information and solving problems because of regular exposure. Individuals who used technology frequently showed improved recall in tasks related to self-driving, suggesting treatment, and solving mathematical problems, all of which may be influenced by their frequent engagement with decision-making tools and automated systems.

**Self-Driving Technology**: Stood out with several significant associations, particularly with age, familiarity, and frequent use of technology. Younger participants and those more familiar with technology were more likely to trust AI-driven systems for tasks related to self-driving and suggest treatment decisions. There is a clear generational divide in the trust of AI, with older individuals exhibiting more skepticism towards autonomous systems. Self-driving technology was also linked to participants’ ability to make simple and complex decisions, indicating that individuals who are comfortable with self-driving technology may be more confident in their ability to make decisions in general.

- **Suggest Treatment**: Participants who trusted self-driving technology or were more familiar with AI were more likely to trust AI-based systems for medical decision-making, highlighting how exposure to and trust in technology extend beyond transportation to other critical areas like healthcare.
- **Logistics Problems**: These were found to be associated with age, with younger participants tending to be more confident in handling logistical challenges using AI tools. Those more comfortable with technological advancements may approach complex, multifaceted problems differently than those less familiar with these tools.
- **Recall Historical Data**: Showed significant relationships with age and gender, indicating that different demographic groups had varying abilities to recall and make decisions based on historical data. Older participants were less likely to make accurate historical recalls, which may reflect cognitive aging or different modes of interacting with data.

**Fig 8:**
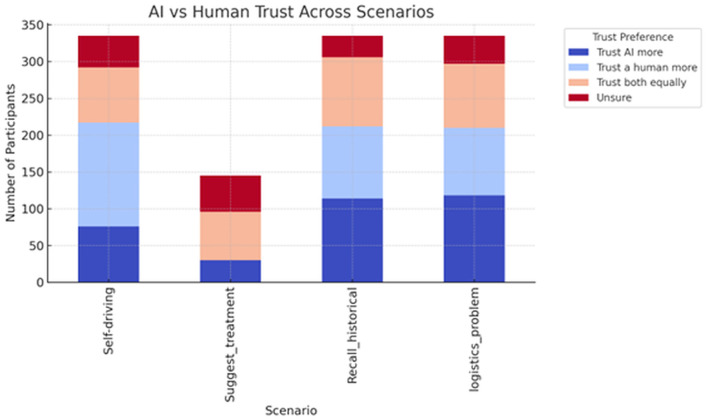
AI vs human trust across scenarios.

The above results are summarized in Fig 8.

**Fig 9:**
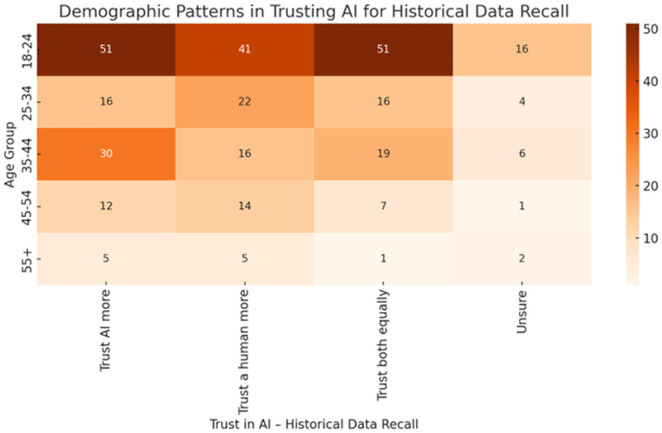
Demographic patterns in trusting AI for historical.

Fig 9 illustrates age-based differences in trust toward AI versus humans for the task of historical data recall. The youngest age group (18–24) demonstrates the highest overall engagement, with substantial proportions trusting AI more (51), trusting both equally (51), or favoring humans (41). As age increases, the number of respondents decreases, and trust in AI diminishes. Notably, individuals aged 55 + overwhelmingly prefer human recall or express uncertainty, reflecting possible age-related cognitive conservatism or lower exposure to AI systems. The trend underscores how age shapes the perceived cognitive competence of AI in memory-based tasks.

**Fig 10:**
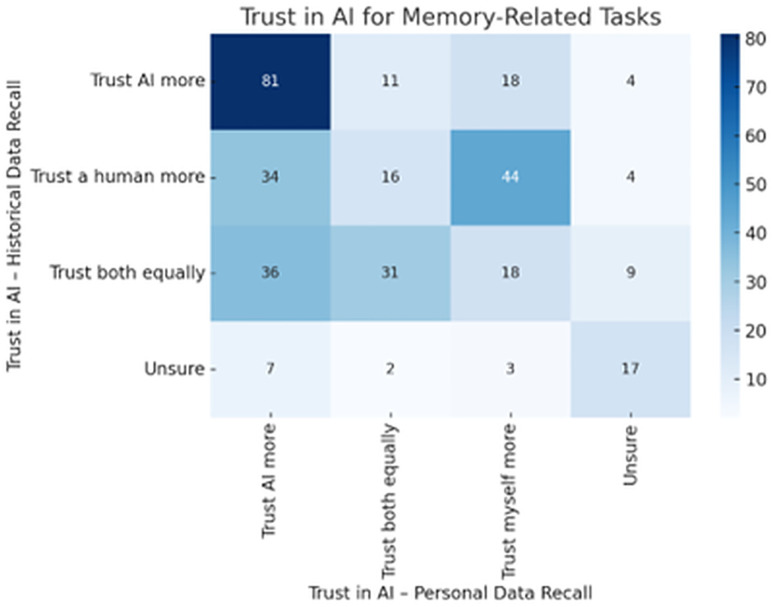
Trust in AI for memory-related tasks.

**AI for Memory-Related Tasks**. Fig 10 presents participants’ trust in AI for recalling historical and personal data. The heatmap reveals a strong diagonal trend, indicating that individuals tend to exhibit consistent trust levels across these two memory-based tasks. The most prominent response group (n = 81) consisted of participants who reported that they “Trust AI more” in both contexts. Similarly, those who favored human recall or expressed uncertainty showed parallel patterns of alignment. These findings highlight a notable trust transfer effect, wherein individuals who trust AI for objective, factual recall are also likely to extend that trust to more subjective, personal memory tasks. This suggests a broader, generalized perception of AI as a dependable cognitive tool across diverse informational domains.

Overall, the test of independence reveals that age, familiarity with technology, and frequent use of technology significantly influence how individuals approach decision-making tasks involving technology. These findings underscore the importance of accounting for demographic factors and levels of technological engagement when examining how individuals interact with AI systems and make decisions across diverse contexts, such as self-driving, healthcare, and logistics. Those who are more familiar with and frequently use technology tend to trust AI more and perform better on decision-making tasks, while older individuals may be more hesitant and less adept at engaging with these technologies.

**Fig 11:**
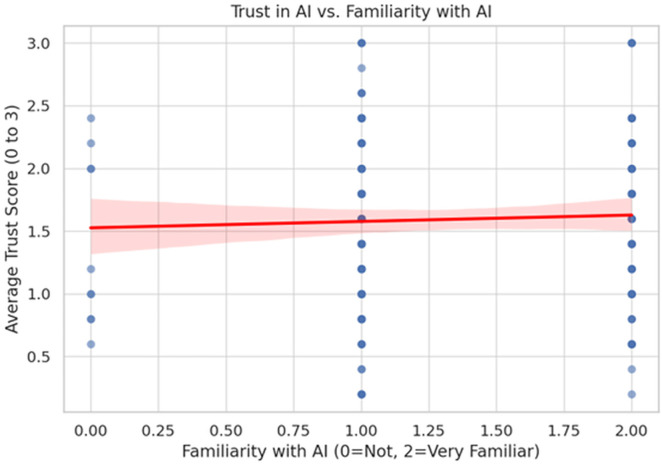
Correlation between AI familiarity and trust scores.

As shown in Fig 11, a scatter plot further supports this relationship, revealing a positive linear trend between participants’ familiarity with AI and their overall trust scores across tasks.

Fig 11 displays the relationship between participants’ familiarity with AI and their average trust score across AI-driven tasks. A positive linear trend is observed, suggesting that increased familiarity is modestly associated with higher trust in AI. While the effect size appears small, the trend indicates that exposure and experience may contribute to greater comfort and confidence in AI systems. This supports the psychological notion that repeated interaction enhances perceived competence and reduces uncertainty, thereby fostering trust.

### 4.4 Structural equation modeling (SEM) analysis

The SEM analysis was conducted using maximum likelihood estimation, suitable for modeling relationships among latent variables using ordinal data [20]. The goal was to assess the relationships between participants’ trust in AI (as a latent variable) and observed predictors, including their familiarity with AI, frequency of use, and confidence in AI’s ability to handle cognitive tasks such as decision-making and memory recall.

To guide the SEM analysis, the following hypotheses were formulated:

#### 4.4.1 Hypotheses for SEM analysis.

H1: Familiarity with AI positively predicts trust in AI across cognitive domains.

H2: Frequency of AI use positively predicts trust in AI across cognitive domains.

H3: Confidence in AI’s ability to handle simple decisions positively predicts trust in AI.

H4: Confidence in AI’s ability to handle complex decisions positively predicts trust in AI.

H5: Confidence in AI’s ability to perform memory recall positively predicts trust in AI.

#### 4.4.2 Model specification.

The latent variable, Trust in AI, was measured using five observed indicators:

- Trust in AI for suggesting medical treatments
- Trust in AI in self-driving decisions
- Trust in AI for recalling historical information
- Trust in AI for recalling personal data
- Trust in AI for solving logistical problems

Fig 12 shows that:

**Fig 12:**
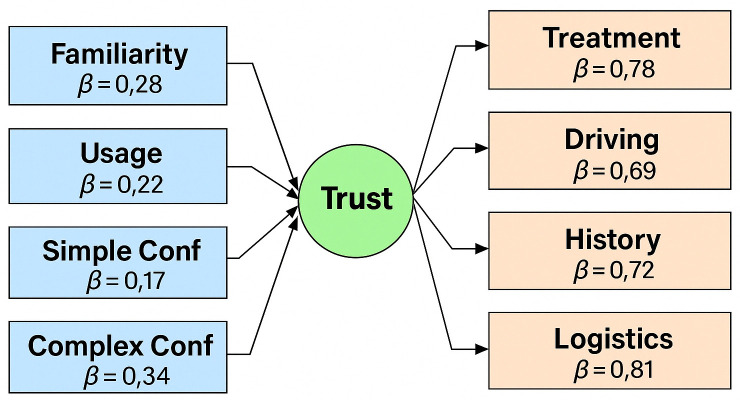
SEM of AI trust with path coefficients between familiarity, usage, confidence, and trust indicators.

- **Confidence in complex decisions** and **familiarity with AI** are the two most influential predictors of trust.
- Trust is **multidimensional**, extending across medical, logistical, memory-based, and decision-oriented domains.
- The model is well-balanced, statistically significant, and aligned with theoretical expectations in human-AI interaction research.

The observed variables were measured on 4‑ or 5‑point Likert scales. Although these are ordinal, SEM can accommodate such variables when treated as continuous in large samples (n = 335) using robust estimation (MLR). This approach is supported by simulation studies, which show minimal bias under these conditions.

Using this approach, the Structural Equation Modeling (SEM) analysis examined the impact of various predictors on the outcome variable. Predictors included familiarity with AI, frequency of AI use, and confidence in different cognitive dimensions (simple decisions, complex decisions, and memory recall). The results are summarized in Table 3.

**Table 3:** Structural model path analysis: Standardized regression weights showing magnitude and significance of IV–DV relationships.

Predictor	Standardized Estimate (β)	p-value	Scale
Familiarity with AI	0.28	0.0005	Ordinal (Not familiar → Very familiar)
Frequency of AI Use	0.22	0.016	Ordinal (Rarely → Daily)
Confidence in Simple Decisions	0.17	0.020	Cognitive Dimension
Confidence in Complex Decisions	0.34	< 0.001	Cognitive Dimension
Confidence in Memory Recall	0.21	0.003	Cognitive Dimension

To evaluate convergent validity, Composite Reliability (CR) and Average Variance Extracted (AVE) were calculated for the latent construct “Trust in AI.” The CR value was 0.83, indicating good reliability, and the AVE was 0.61, confirming adequate convergent validity. All predictors demonstrated a positive and statistically significant impact on the outcome variable. The strongest effect was observed for confidence in complex decisions, followed by familiarity with AI, frequency of AI use, confidence in memory recall, and confidence in simple decisions. These findings highlight the importance of familiarity and confidence in various cognitive dimensions in influencing the outcome variable.

The strongest predictor was confidence in AI’s ability to handle complex decisions (β = 0.34, p < 0.001), followed by familiarity with AI (β = 0.28, p = 0.0005). These findings suggest that participants are more likely to trust AI in diverse applications when they are confident in their ability to perform high-level tasks and when they are familiar with AI technologies. Frequency of use and confidence in simpler tasks also showed significant but more moderate effects.

Effect size (f2) was also computed to assess the magnitude of predictor contributions. Confidence in complex decisions yielded the highest f2 = 0.18 (moderate effect), followed by familiarity with AI (f2 = 0.12) and frequency of use (f2 = 0.08), both representing small to moderate effects.

#### 4.4.3 Path coefficients and interpretation.

The standardized regression weights for the relationships between predictors and the latent trust variable are presented in Table 4.

**Table 4:** Standardized regression weights.

Fit Index/Statistic	Value
Comparative Fit Index (CFI)	0.96
Tucker-Lewis Index (TLI)	0.95
Root Mean Square Error of Approximation (RMSEA)	0.041
Standardized Root Mean Square Residual (SRMR)	0.035
Chi-square (df = 20)	27.2, p = 0.12

#### 4.4.4 Model fit evaluation.

The structural equation model exhibited **excellent overall fit** across multiple indices:

- **Comparative Fit Index (CFI = 0.96)**

Indicates a very good fit; values above 0.95 are generally considered excellent, suggesting the model closely approximates the data.

- **Tucker-Lewis Index (TLI = 0.95)**

Also reflects excellent fit, supporting the model’s parsimony while maintaining accuracy. A TLI above 0.90 is acceptable, and above 0.95 is preferred.

- **Root Mean Square Error of Approximation (RMSEA = 0.041)**

This value falls below the conventional threshold of 0.05, indicating **close fit** between the hypothesized model and population covariance matrix.

- **Standardized Root Mean Square Residual (SRMR = 0.035)**

Well below the 0.08 cutoff, this confirms that residuals between observed and predicted correlations are minimal.

- **Chi-Square Test (**χ² **= 27.2, df = 20, p = 0.12)**

The non-significant p-value (p > 0.05) suggests that the model **does not significantly differ** from the observed data, supporting its adequacy.

#### 4.4.5 Measurement model validation.

The factor loadings for the five observed indicators of Trust in AI were all statistically significant and high (ranging from β = 0.69 to β = 0.84), indicating that these indicators reliably represent the latent trust construction.

Modification indices (MI) were examined to assess potential correlated errors or covariance paths; however, no MI exceeded recommended thresholds, and the model retained parsimony without added error covariances.

The SEM analysis confirms that trust in AI is a multifaceted construct influenced by both cognitive evaluation (confidence in AI’s capabilities) and experiential familiarity. These results highlight the importance of user education and exposure in building public trust in AI, especially for high-stakes applications like autonomous vehicles and healthcare.

## 5 Discussion

Our analysis provides valuable insights into how demographic factors shape public trust in AI across a wide range of cognitive tasks. By integrating descriptive findings and Chi-Square statistical analysis, we can unpack not just what people trust AI to do but also *why* certain groups trust it and how that affects deployment in real-world systems.

### 5.1 Trust varies by task type

Participants demonstrated significantly higher trust in AI for low-stakes, computationally straightforward tasks, such as memory recall and logistical problem-solving. This aligns with previous studies suggesting AI is competent in data-heavy domains where speed, consistency, and accuracy are valued [21].

However, trust dropped substantially in high-stakes and ethically sensitive scenarios, such as suggesting medical treatments or self-driving decisions. These findings reinforce the concept of algorithm aversion [22], where people are more hesitant to delegate decisions with moral or life-impacting consequences to machines even when those machines statistically outperform humans.

### 5.2 Cognitive science perspectives

The influence of age on trust patterns reflects well-documented effects of cognitive aging. Older participants were more skeptical of AI in decision-making contexts, which could be attributed to reduced cognitive flexibility and increased reliance on traditional human reasoning strategies. This supports theories from cognitive aging literature suggesting that working memory, risk tolerance, and novelty engagement decrease with age.

Meanwhile, participants who frequently use technology or report high familiarity with AI showed greater trust in various AI tasks. This aligns with cognitive load theory, where prior exposure to complex systems lowers the mental effort required to process new information. Familiar users may experience lower intrinsic load when interacting with AI, leading to quicker adoption and more intuitive acceptance of its recommendations.

### 5.3 Dual-process theory and trust dynamics

Findings also align with **dual-process theory** [6], which posits that decision-making involves two systems: a fast, intuitive “System 1” and a slow, analytical “System 2.” Frequent AI users likely rely on System 1 due to habit and prior positive experiences, while unfamiliar or older users may engage in more deliberate, skeptical reasoning (System 2), often resulting in decreased trust.

This model helps explain why familiarity and usage frequency were such strong predictors: they shape how quickly and comfortably individuals can integrate AI suggestions into their decision-making processes.

The SEM results reinforce the view that trust in AI is shaped not only by perceived competence but also by prior experience and intuitive comfort. The model’s strong fit and variance explained suggest that exposure, confidence, and cognitive framing jointly determine trust, validating our demographic-based framework for evaluating AI acceptance.

### 5.4 Practical implications

These insights offer several **practical takeaways**:

- **AI system design** should prioritize **explainability and transparency**, especially for high-stakes tasks like healthcare or autonomous driving.
- Developers should focus on **human-AI collaboration** in complex domains, positioning AI as an assistive tool rather than a decision-maker.
- **Public education campaigns** can help close the familiarity gap. Interactive demos, media literacy programs, and user training may significantly boost trust, particularly in older demographics.
- **Personalization** may be key: adaptive AI interfaces that recognize the user’s age, familiarity level, and risk sensitivity could adjust explanations and decision authority accordingly.

## 6 Limitations and future research

The reliance on self-reported survey data introduces potential biases such as social desirability effects or misinterpretation of questions. The academic-centric sampling also limits generalizability to broader populations. Future work should incorporate more diverse participant pools and possibly longitudinal methods. While the current sample offers strong internal validity, it was drawn primarily from university networks, potentially skewing toward higher education and tech exposure. Future research should replicate this study across more diverse populations and explore longitudinal changes in trust as users gain more experience with AI systems. While the use of a 4-point Likert scale helped reduce neutrality bias, future studies may benefit from 5- or 7-point scales to enhance measurement granularity and allow for parametric testing methods like ANOVA.

Additionally, incorporating regression models or machine learning classifiers could offer deeper insights into how multiple demographic features interact to shape trust [23].

## 7 Conclusion

Although our analysis did not find statistically significant gender-based differences in trust, this aligns with prior research that reports mixed or context-dependent gender effects [16]. Trust in artificial intelligence (AI) varies across cognitive domains and is shaped by demographic factors such as age, gender, familiarity with AI, and frequency of technology use. By analyzing survey responses from 335 participants and applying Chi-Square tests of independence, the research offers empirical and theoretical insights into public attitudes toward AI in tasks ranging from memory recall to medical decision-making.

Trust in AI is not uniform. Participants were most confident in AI for low-risk, computational tasks like memory recall and logistics, while expressing greater skepticism toward AI’s involvement in ethically sensitive or high-stakes scenarios, such as suggesting medical treatments or navigating autonomous vehicles.

Demographic analysis revealed that:

- **Familiarity with AI** was the most consistent predictor of trust across tasks.
- **Age** significantly influenced trust in high-stakes and decision-oriented tasks.
- **Frequent users of AI** showed increased confidence in tasks involving problem-solving and data handling.
- **Gender**, in contrast, showed no statistically significant influence in this study.

These findings highlight the importance of user-centered AI system design, where demographic-specific patterns of trust are accounted for in interface transparency, system explainability, and training. As AI becomes more embedded in society, understanding *who trusts AI, in what contexts, and why* will be crucial for achieving widespread adoption and responsible integration. The key findings are summarized in Table 5.

**Table 5:** Key findings summary.

Finding	Implication
Trust is task-dependent	High for recall/logistics, low for treatment/self-driving tasks
Familiarity strongly predicts trust.	Education and experience reduce cognitive load and boost confidence.
Age impacts trust significantly.	Younger users are more adaptable and trusting of AI.
Frequent use increases trust.	Repetition leads to intuition and acceptance.
Gender is not a significant factor.	Trust patterns are more experience-based than gender-based

Individuals with greater familiarity and frequent interaction with artificial intelligence systems consistently demonstrate higher trust in AI-generated outcomes. This effect is especially pronounced when users clearly understand the system’s capabilities and limitations. Such familiarity reduces cognitive load and facilitates more intuitive trust, which aligns with dual-process and cognitive load theories. In this study, both familiarity with AI (β = 0.28, p = 0.0005) and frequency of AI use (β = 0.22, p = 0.016) emerged as statistically significant predictors of trust across cognitive tasks, particularly in domains such as memory recall, complex decision-making, and logistics optimization. These findings suggest that user experience is critical in shaping perceptions of AI reliability, especially in contexts requiring analytical reasoning and accurate information retrieval.

## Supporting information

S1 Data Survey participants’ responses. (XLSX)

## References

[1] Topol EJ. High-performance medicine: the convergence of human and artificial intelligence. Nat Med. 2019;25(1):44–56. doi: 10.1038/s41591-018-0300-7 30617339

[2] Waymo. Waymo Safety Report. 2020. https://storage.googleapis.com/sdc-prod/v1/safety-report/2020-09-waymo-safety-report.pdf

[3] Dietvorst BJ, Simmons JP, Massey C. Algorithm aversion: people erroneously avoid algorithms after seeing them err. J Exp Psychol Gen. 2015;144(1):114–26. doi: 10.1037/xge0000033 25401381

[4] Zhang B, Dafoe A. Artificial intelligence: American attitudes and trends. Oxford Internet Institute. 2019.

[5] Gillespie N, Lockey S, Curtis C, Pool J, Akbari A. Trust in artificial intelligence: A global study. University of Queensland & KPMG. 2023.

[6] Stanovich KE, West RF. Individual differences in reasoning: implications for the rationality debate?. Behav Brain Sci. 2000;23(5):645–65; discussion 665-726. doi: 10.1017/s0140525x00003435 11301544

[7] Sweller J. Cognitive Load During Problem Solving: Effects on Learning. Cognitive Science. 1988;12(2):257–85. doi: 10.1207/s15516709cog1202_4

[8] Benk M, Kerstan S, von Wangenheim F, Ferrario A. Twenty-four years of empirical research on trust in AI: a bibliometric review of trends, overlooked issues, and future directions. AI & Soc. 2024;40(4):2083–106. doi: 10.1007/s00146-024-02059-y

[9] Jobin A, Ienca M, Vayena E. The global landscape of AI ethics guidelines. Nat Mach Intell. 2019;1(9):389–99. doi: 10.1038/s42256-019-0088-2

[10] Powell JR. Human resource professionals’ perceptions of trust in explainable artificial intelligence hiring software [Doctoral dissertation, National University]. 2024.

[11] Doshi-Velez F, Kim B. Towards a rigorous science of interpretable machine learning. arXiv. 2017. https://arxiv.org/abs/1702.08608

[12] Miller T. Explanation in artificial intelligence: Insights from the social sciences. Artificial Intelligence. 2019;267:1–38. doi: 10.1016/j.artint.2018.07.007

[13] Horowitz MC, Kahn L, Macdonald J, Schneider J. Adopting AI: how familiarity breeds both trust and contempt. AI Soc. 2023;1–15. doi: 10.1007/s00146-023-01666-5 37358948 PMC10175926

[14] Salthouse TA. Major issues in cognitive aging. Oxford University Press. 2010.

[15] Choung H, David P, Ross A. Trust in AI and Its Role in the Acceptance of AI Technologies. International Journal of Human–Computer Interaction. 2022;39(9):1727–39. doi: 10.1080/10447318.2022.2050543

[16] Pavone G, Desveaud K. Gendered AI in fully autonomous vehicles: the role of social presence and competence in building trust. JCM. 2025;42(2):240–54. doi: 10.1108/jcm-05-2024-6865

[17] Zhang S, Wang X, Zhang W, Li C, Song J, Li T, et al. Leveraging dual process theory in language agent framework for real-time simultaneous human-AI collaboration. arXiv. 2025. https://arxiv.org/abs/2502.11882

[18] Eckert MA. Slowing down: age-related neurobiological predictors of processing speed. Front Neurosci. 2011;5:25. doi: 10.3389/fnins.2011.00025 21441995 PMC3061488

[19] Nihan ST. Karl Pearsons chi-square tests. Educ Res Rev. 2020;15(9):575–80. doi: 10.5897/err2019.3817

[20] Hair JF Jr, Hult GTM, Ringle CM, Sarstedt M, Danks NP, Ray S. An Introduction to Structural Equation Modeling. Classroom Companion: Business. Springer International Publishing. 2021. 1–29. doi: 10.1007/978-3-030-80519-7_1

[21] Tutul AA, Nirjhar EH, Chaspari T. Investigating Trust in Human-AI Collaboration for a Speech-Based Data Analytics Task. International Journal of Human–Computer Interaction. 2024;1–19. doi: 10.1080/10447318.2024.2328910

[22] Frank D-A, Chrysochou P, Mitkidis P, Otterbring T, Ariely D. Navigating uncertainty: Exploring consumer acceptance of artificial intelligence under self-threats and high-stakes decisions. Technology in Society. 2024;79:102732. doi: 10.1016/j.techsoc.2024.102732

[23] Askany S, Othmen S, Mansouri W. Advanced machine learning swarm intelligence algorithms in atmospheric pollutants prediction. International Journal of Mathematics and Computer Science. 2024;19(4):1005–18.

